# Stage 1 hybrid palliation of hypoplastic left heart syndrome: an initial experience in pulmonary trunk approach, procedural modifications, and complication management

**DOI:** 10.3906/sag-1903-143

**Published:** 2019-10-24

**Authors:** Emrah AKAY, Onur IŞIK, Ayşen Yaprak ENGİN, Volkan ÇAKIR

**Affiliations:** 1 Department of Radiology, Faculty of Medicine, Balıkesir University, Balıkesir Turkey; 2 Department of Pediatric Cardiovascular Surgery, Tepecik Training and Research Hospital, İzmir Turkey; 3 Department of Cardiovascular Surgery, Tepecik Training and Research Hospital, İzmir Turkey; 4 Department of Radiology, Faculty of Medicine, Kâtip Çelebi University, İzmir Turkey

**Keywords:** Pediatrics, interventional vascular, stents, vascular, congenital

## Abstract

**Background/aim:**

Hypoplastic left heart syndrome (HLHS) is a rare pathology with a very high mortality rate. The present study aimed to share our initial experience with the ductus arteriosus stenting procedure using the pulmonary trunk approach in the treatment of HLHS, as well as provide some technical suggestions and discuss complications and their management.

**Materials and methods:**

The medical records of 9 neonates (age range: 1–8 days) with HLHS, who were operated on within a 12-month period, were reviewed retrospectively. Preprocedural planning was performed by computed tomography angiography and echocardiography. The operations were performed in a hybrid surgery room by interventional radiologists and pediatric vascular surgeons. Balloon-expandable stents were used in all of the operations.

**Results:**

All operations were successfully completed without any intraoperative mortality. All intraoperative complications were managed successfully during the stenting procedure.

**Conclusion:**

Stage 1 hybrid palliation for HLHS is a safe and effective procedure when several key points are kept in mind.

## 1. Introduction

Hypoplastic left heart syndrome (HLHS), which was first described by Lev in 1952 as hypoplasia of the aortic tract complexes, is characterized by hypoplasia of the aortic arcus and developmental failure of the left-sided heart chambers at varying degrees [1]. Inadequate treatment of this pathology results in a decrease in life expectancy to 10% within the first month of life [2]. Prenatal diagnosis of HLHS enables the early infusion of prostaglandin, which in turn facilitates the maintenance of systemic cardiac output through the ductus arteriosus (DA) and improves survival rates after the first stage of palliative surgery when compared with postnatal diagnoses. [3].

Stage 1 hybrid palliation, which was first described by Gibbs et al. [4] in 1993, consists of bilateral branch pulmonary artery banding and stenting of the DA without cardiopulmonary bypass during the neonatal period. With recent advances in endovascular interventional technology, hybrid palliation has become popular as an alternative to the stage 1 Norwood procedure. This procedure can also be used as an alternative to pretransplant palliation and salvage of the patients [5]. Hybrid palliation mainly aimed at controlling blood flow through the pulmonary arteries, preserving coronary blood flow, and obtaining optimum cardiac output and systemic blood flow while maintaining an unrestricted atrial septum. The main advantage of hybrid palliation over the Norwood 1 procedure is the fact that it provides the opportunity to avoid cardiopulmonary bypass and cardioplegic arrest during the neonatal period [6].

The present study aimed to present the procedural outcomes of the stage 1 hybrid palliation and pulmonary trunk approach in the treatment of HLHS, as well as provide some practical key points and discuss complications and their management using the modified technical approach for stenting of the DA.

## 2. Materials and methods

In the present study, the medical records of 9 neonates who underwent stage 1 hybrid palliation for HLHS between September 2015 and September 2016 were retrospectively reviewed. The ages of the neonates ranged from 1 to 8 days, the mean age was 5 days, and 3 of the neonates were premature. Under prostaglandin E1 infusion, the preoperative blood oxygen saturation levels were between 87% and 98%, and the average saturation was 91%. While one neonate presented with accompanying type B aortic interruption and right ventricular hypoplasia, there was no accompanying aorta coarctation diagnosed in the patient group.

The study was approved by an institutional ethical committee and informed consent was obtained from the parents of all neonates after providing details about the procedure and associated potential complications.

Performing a hybrid palliation procedure for each patient was decided by consensus at meetings including a congenital cardiovascular surgeon, a pediatric cardiologist, and an interventional radiologist. Preprocedural planning was primarily performed according to the images obtained by computed tomography (CT) angiography using a 128-slice CT device (Siemens SOMATOM Definition AS, Siemens AG, Germany). CT angiographic images provide a substantial amount of information about the anatomy; however, as marked changes occur in the DA caliber within a short time frame, the DA was reevaluated echocardiographically (Phillips HD 11 XE, Amsterdam, the Netherlands) immediately before the procedure to observe any changes in the DA diameter.

All of the operations were performed under general anesthesia in a hybrid operating room using a floormounted monoplane C-Arm system (Artis Zee, Siemens Healthcare GmbH, Erlangen, Germany). The neonates were monitored using a 5-lead electrocardiogram, an arterial line inserted into the left radial artery, and a 4-French venous central line in the right internal jugular vein, as well as a capnometer for the measurement of carbon dioxide levels and a peripheral oximeter for the measurement of oxygen saturation. After preparation of the skin at the supine decubitus position, a median upper partial sternotomy incision was performed and the thymus was resected. Following the pericardial incision, bilateral pulmonary artery banding was performed. The bands were prepared using a 3.5-mm polytetrafluoroethylene tube graft. A circular 6-0 Prolene (Ethicon, São Paulo, Brazil) purse string suture was placed around the designated puncture site at the main pulmonary trunk.

**Figure 1:**
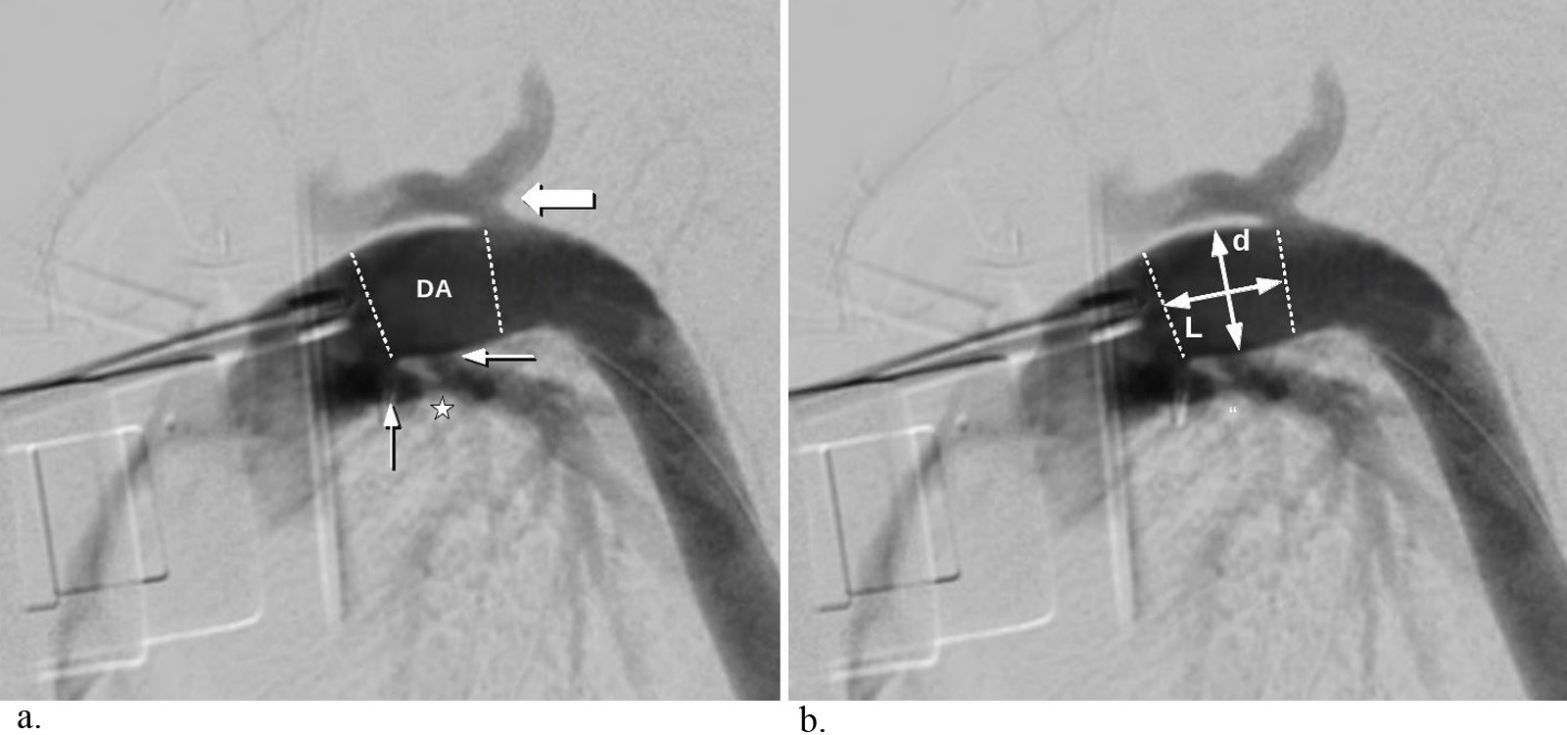
A lateral oblique angiographic image used to determine critical structures and stent size. (a) The dotted lines indicate the borders of the ductus arteriosus (DA). The star indicates the indentation from the graft bands over the pulmonary arteries shown by the thin arrows. The retrograde contrast flow (marked with a bold arrow) demonstrates the insertion of the native arcus. (b) The measurements for the stent are marked as L and d, where L indicates the proper length and d indicates the diameter.

After sheath placement, 50 IU/kg of heparin was administered and angiographic images were acquired using diluted contrast material (25% contrast + 75% saline; Ultravist 370/100 Bayer Schering Pharma AG, Berlin, Germany). The proximal and distal parts of the DA, DA caliber, caliber of the pulmonary arteries, and native arcus junction segment were marked (Figure 1a). A 0.014-inch guidewire, at a standard length of 180 cm (Asahi Intecc Co., Ltd., Aichi, Japan), was used with a rapid exchange system. An appropriately sized stent (Palmaz Blue .014 Peripheral Stent System, Cordis Medical, Switzerland) was confirmed after measurement of the dimensions of the DA on the left lateral oblique projection (Figure 1b). The diameters and lengths of the used stents ranged from 5 mm to 10 mm and from 8 mm to 15 mm, respectively. The mean diameter of the DA was 7 mm. During stent deployment, balloon inflation and deflation procedures were performed promptly to avoid prolonged increased cardiac afterload. After stent deployment, control angiograms were acquired and appropriate stent placement was verified. Prostaglandin E1 infusion was then discontinued.

After the procedure was completed, the neonates were transferred to the neonatal intensive care unit. The neonates were extubated following 12 h of ventilation; the oxygen saturation was continuously monitored and heparin infusion at a rate of 25 units/kg/h was continued for 72 h.

## 3. Results

All of the hybrid procedures were completed successfully without any intraoperative mortality. Blood oxygen saturation levels after cessation of the prostaglandin E1 infusion were between 83% and 95%, and the average saturation level was 87%. Two of the neonates (n = 9) died within 60 days after the surgery. One of the neonates died due to the occurrence of septicemia following catheter angiography for atrial septostomy and the other neonate died due to stent migration during septal balloon angioplasty. Within a mean period of 30 days (range: 16– 60 days), 7 patients were discharged from the hospital and were waiting for the second-stage treatment (Norwood– Glenn procedure) at the time of completion of this study.

## 4. Discussion

The surgical methodology of bilateral pulmonary artery banding and ductal stenting has been well documented in many different publications [4,6,7]; however, the endovascular technique has not been fully documented for the pulmonary artery approach. Accordingly, herein, its various associated complications and key facts about its management will be discussed from an interventional radiologist perspective.

**Figure 2:**
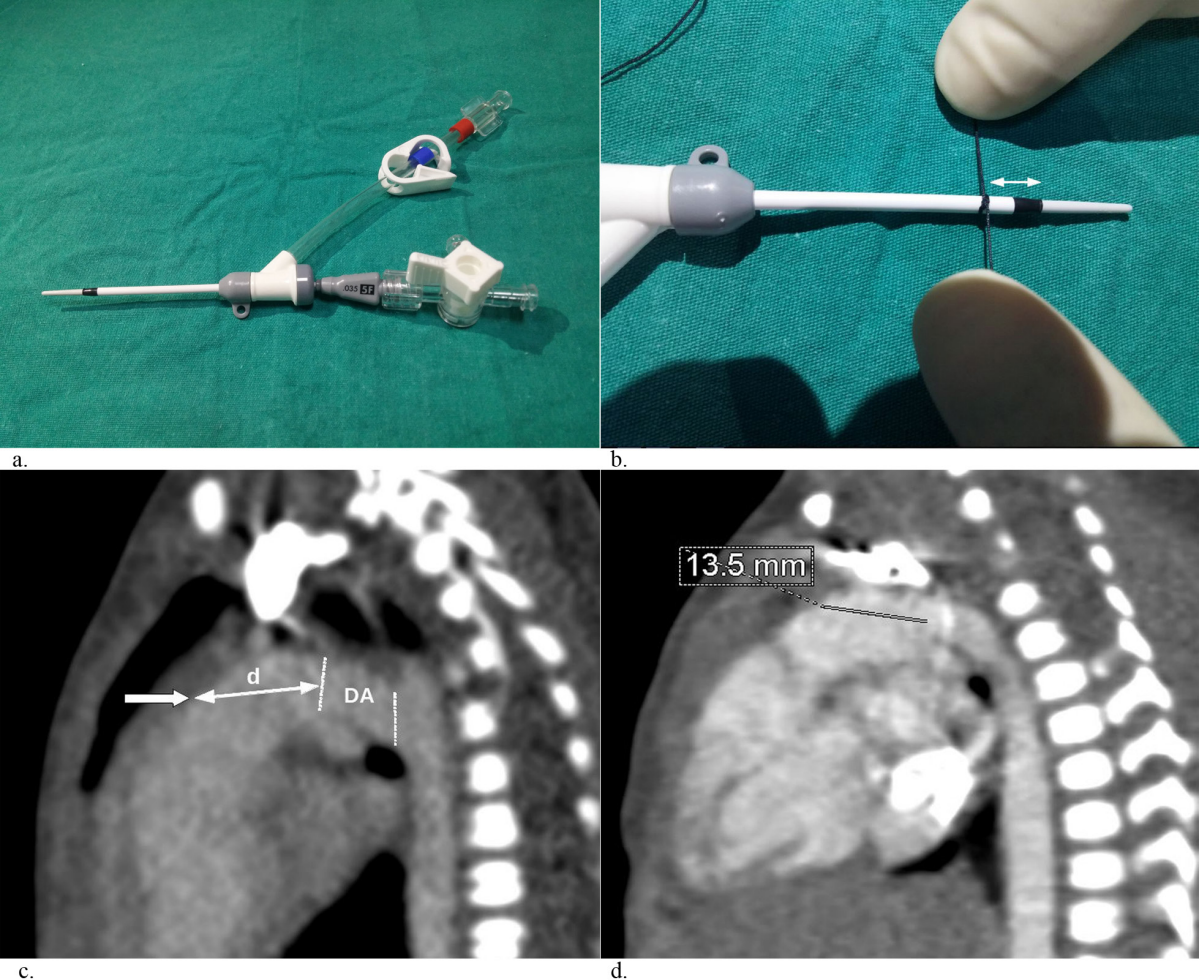
Selection and preparation of the introducer sheath. (a) The length of a short shaft, a distal radio-opaque marker, a smooth sheath-to-dilator transition, and a side-port with a snap clamp to allow for introducing a buddy-wire if needed. (b) Image of application of a security knot on the introducer sheath shaft. The distance (indicated by the double-headed arrow) from the knot to the sheath tip is measured using computed tomography (CT) images. (c) The sagittal CT image showing the measurement of the security knot distance d; the bold arrow indicates the designated puncture site and the dotted lines indicate the DA. (d) Markedly short puncture distance to DA is a major problem in premature neonates. The CT image of a case in which the puncture distance was measured as 13.5 mm, meaning that only 1 cm of the sheath segment can be introduced inside the pulmonary trunk.

During the procedure, a standard 10-cm introducer sheath was not used due to difficulty in manipulating a long sheath and its compatibility with 0.035-inch guidewires. Although these sheaths can be advanced over 0.014–0.018- inch guidewires, the potential gap between the guidewire and the lumen of the dilator could possibly lead to the initiation of a dissection in the pulmonary trunk or in the DA during sheath placement. Short 5-F radial introducers, which are 7 cm in length and compatible with 0.018-inch guidewire, and a distal radio-opaque marker were used (Figure 2a). Difficulty identifying the tip of the sheath was experienced without a distal marker, which resulted in sheath displacement and accidental stent deployment inside the sheath.

Currently, both self- and balloon-expandable stents are used for DA stenting [8]. Balloon-expandable stents are considered to have several evident advantages over self-expandable types. While the shape-memory property of self-expandable stents may prove to be useful in tortuous DAs, the precise deployment of a balloonexpandable stent, especially under a high blood volume state in a short segment, is superior to precise deployment of a self-expandable stent. Moreover, in the event of stent migration, the manipulation and redeployment of a balloon-expandable stent is easier. Uncovered DA segments may lead to a coarctation and result in restricted coronary or systemic blood flow [9]. Development of a coarctation, which is a long-term complication, can be prevented by selecting an appropriately sized stent that will cover the ductus as much as possible.

One of the main problems encountered in the pulmonary artery approach was the difficulty of holding the introducer sheath in place during the whole stenting procedure. Advancing the sheath too much would cause difficulty in precise placement of the stent, in addition to a potential dissection induced by the tip of the sheath. On the other hand, retracting the sheath too much would cause bleeding complications and possible loss of access. Accordingly, the problem was solved by applying a security knot around the distal end of the sheath body (Figure 2b). This knot served as a barrier to avoid over advancement of the sheath inside the artery. As this barrier approached the arteriotomy site, it prevented inadvertent movement of the sheath and made stabilization of the sheath in its place easier during the procedure. This method enabled the avoidance of serious complications, such as loss of access and dissection. The position of the suture was determined on the sagittal CT angiography images by measuring the distance from the designated puncture site to the beginning of the DA (Figure 2c).

During the initial puncture, the guidewire was kept ready inside the needle to allow its quick advancement within the pulmonary artery. Advancing the floppy segment of the guidewire inside of the hub of the needle immediately after the initial puncture may be difficult due to the high-velocity pulsatile blood flow from the needle lumen. The standard short guidewires commercially packaged with the sheaths were not used during the initial placement of the introducer sheaths. Instead, the procedure was initiated using a standard 180-cm, 0.014- inch guidewire, and the guidewire was advanced until the floppy segment reached the level of external iliac artery. By securing the guidewire at this level, the operator had the possibility of avoiding complications at the puncture site during the procedure, and it was ensured that the position of the stent would be preserved in its place over the guidewire in the event of stent migration.

### 4.1. Complications and their management

#### 4.1.1. Loss of access

Working on a continuously moving vascular structure inside a tight sternotomy area, operators may find themselves struggling to hold the introducer in place during the procedure. After the initial applications, it was realized that an assistant surgeon needed to hold the sheath and the purse suture in place throughout the intervention. In case of loss of access, a new sheath was advanced over the guidewire through the arteriotomy site while the assistant surgeon tightened the purse suture for bleeding control. When both the sheath and guidewire were lost, it was proven to be ideal to reposition the sheath through the prior arteriotomy site using a mounted dilator.

#### 4.1.2. Deformation of the introducer tip and access site rupture

Tip deformation occurs when the introducer sheath encounters abnormally high resistance while entering the artery. This occurs when the weakening procedure is applied in a suboptimal manner or when the weakening procedure is not applied all over the muscular layers of the designated puncture site on the pulmonary trunk. While the dilator passes through the artery wall effortlessly, an excessive resistance at the dilator-body junction may cause structural deformation at the introducer tip. If the operator fails to notice this condition and forces the sheath into the artery, an arterial wall rupture may occur at the puncture site.

#### 4.1.3. Dissection

**Figure 3:**
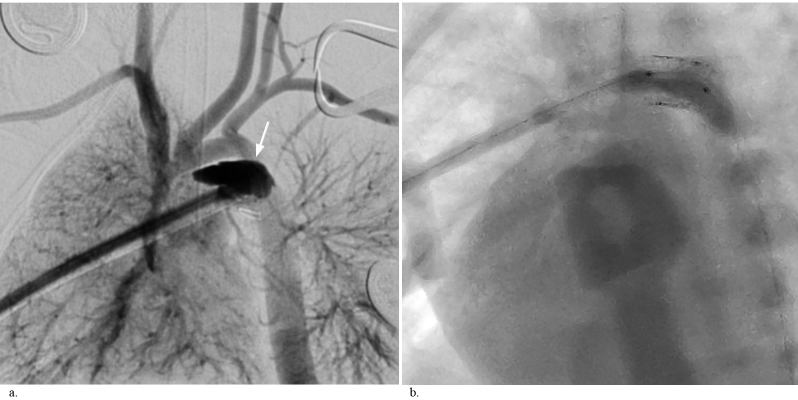
DA dissection. (a) Appearance of the dissection flap as contrast retention after the injection. Note the dissection from the midline to the end of the DA caused by accidental advancement of the introducer sheath inside the DA. This was the first and only case in which a long shaft sheath without a security knot was used. (b) Image of the urgent stent deployment over the dissected segment.

It was observed that uncontrolled movement of the introducer sheath caused endothelial injury in the DA. In the absence of a mounted dilator, the tip of an introducer becomes a relatively stiff and sharp structure. Holding the introducer steady in its place is a challenge, particularly in premature cases, in which the distance between the puncture site and the DA is considerably small (Figure 2d), and minute movements may initiate a dissection. This could be overcome by applying a security knot. A dissection can be recognized based on the view of residual contrast material with a slit-like appearance at the apex of the DA on the angiograms (Figure 3a). The angiogram image may indicate an early-stage dissection plane and contrast filling of the false lumen. This type of dissection will tend to be flow-restricting, as the intimal flap will face the flow direction.

A dissection, which progressed to acute heart failure, due to a rapid increase in the cardiac afterload, occurred in one of the neonates. Cardiac arrest occurred within a 1-min period, and the neonate did not respond to resuscitation attempts until a stent was implemented urgently over the dissection site (Figure 3b).

#### 4.1.4. Stent migration

There are several reported alternative ways of dealing with a migrated stent in the medical literature [10]. A migrated stent can be pulled back using a snare catheter; however, the mesh of a balloon-expandable stent can easily be deformed and may get stuck at the tip of the introducer. Accordingly, it was concluded that the most favorable practice was to pull the stent back to the DA using an angioplasty balloon.

**Figure 4:**
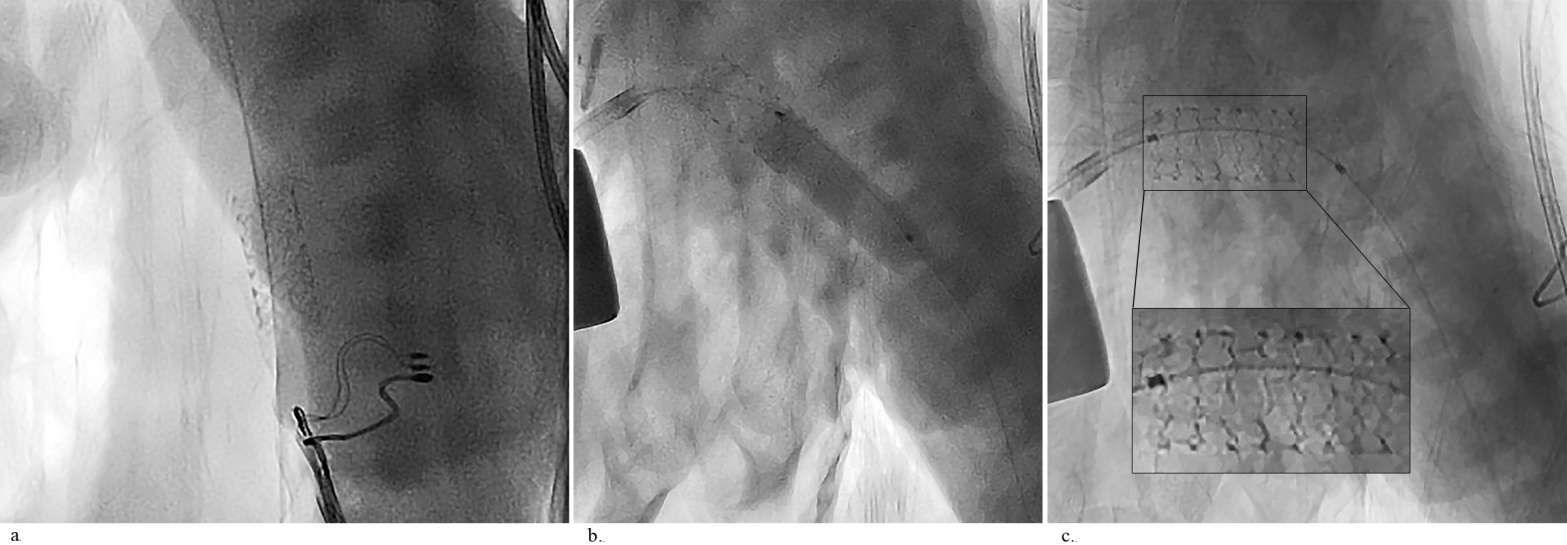
Stent migration. (a) Image of the migrated stent from the DA to the celiac segment of the abdominal aorta. Note that the stent is still over the guidewire that was advanced to the iliac artery after the first puncture, making it easier to retrieve the stent. (b) The stent was pulled back to the DA with a balloon under continuous fluoroscopic guidance. (c) The stent was expanded with a larger balloon. Note the deformed mesh structure of the stent due to oversizing of the magnified image.

In the present study, stent migration was encountered in only one case during the application. Following the replacement of the stent balloon with a larger one, the balloon was advanced and passed the migrated stent at the abdominal aorta (Figure 4a). Next, after ensuring that the markers were outside of the stent, the balloon was inflated submaximally. Under continuous fluoroscopic guidance, the balloon was gradually pulled back until the stent was placed inside the DA (Figure 4b). The distal part of the stent was squeezed partially by forcing the balloon inside it to ensure that the distal meshes came into contact with the vessel wall in order to temporarily stabilize the stent. The balloon was then deflated and repositioned in the center of the stent. Finally, the balloon was inflated maximally to oversize the stent. The angiograms following the procedure revealed that the stent was appropriately placed, whereas the mesh structure was deformed due to oversizing (Figure 4c).

### 4.2. Conclusions

Bilateral pulmonary artery banding and ductal stenting procedures are becoming increasingly popular due to innovative endovascular device technology and improved observable clinical outcomes. Recent studies have suggested that hybrid palliation provides advanced postoperative recovery and similar survival rates when compared with the Norwood palliation method. Moreover, it provides the achievement of preserved ventricular function in stage 2 palliation [11,12].

Previous studies on mortality have reported a survival rate of 80% to 97% after hybrid procedures [2]. The results herein were also similar to those reported in the literature related to early mortality; none of the patients (n = 9) died intraoperatively or in the early postoperative period.

Although there are some specific procedural difficulties with the application of the pulmonary artery approach for ductal stenting, we believe that it also has evident advantages over the transvenous and retrograde femoral artery routes reported in some large series [6,13]. For instance, the transvenous route approach possesses the risk of arrhythmia while advancing the stent through the right heart [13]. Likewise, in the retrograde femoral artery approach, placement of an appropriate-sized sheath into the femoral artery may be challenging and can lead to complications at the puncture site in premature newborns. Our preliminary experience suggested that by keeping some practical key points in mind, ductal stenting using the pulmonary artery approach could be performed safely and effectively.

The current study had some limitations, including its retrospective design and small number of patients. Until further clinical trials demonstrate the benefits of the hybrid palliation approach when compared with conventional Norwood stage 1 surgery, the decision to administer surgical reconstruction or the hybrid approach should be made based on physicians’ individual experience in performing each procedure.
